# Affinity Affects the Functional Potency of Anti-GD2 Antibodies by Target-Mediated Drug Disposition

**DOI:** 10.3390/cancers17152510

**Published:** 2025-07-30

**Authors:** Sascha Troschke-Meurer, Maxi Zumpe, Peter Moritz Ahrenberg, Torsten Ebeling, Nikolai Siebert, Piotr Grabarczyk, Holger N. Lode

**Affiliations:** 1 Department of Pediatric Oncology and Hematology, University Medicine Greifswald, 17475 Greifswald, Germany; sascha.troschke-meurer@med.uni-greifswald.de (S.T.-M.); maxi.zumpe@med.uni-greifswald.de (M.Z.); petermoritz.ahrenberg@med.uni-greifswald.de (P.M.A.); torsten.ebeling@med.uni-greifswald.de (T.E.);; 2 Clinic for Internal Medicine C—Hematology, Oncology, University Medicine Greifswald, 17475 Greifswald, Germany; piotr.grabarczyk@med.uni-greifswald.de

**Keywords:** neuroblastoma, GD2-based immunotherapy, dinutuximab beta, naxitamab, antibody binding affinity, target-mediated drug disposition

## Abstract

Background/Objectives: High-risk neuroblastoma patients are treated with approved anti-ganglioside GD2 antibodies of moderate (dinutuximab beta; DB) and higher binding affinity (naxitamab; NAXI). We evaluated the functional potency of DB compared to NAXI and investigated the target-mediated drug disposition (TMDD). Methods: Tumor spheroids were generated from neuroblastoma cells with varying GD2 expression, stably expressing iRFP680 as a viability marker. Antibody-dependent cellular cytotoxicity (ADCC) and complement-dependent cytotoxicity (CDC) were assessed in a long-term life-cell viability assay using serial dilutions of the GD2 antibodies. Binding activity was determined by flow cytometry. Processes involved in TMDD were analyzed, including antibody binding to dead tumor cells and to soluble GD2 (sGD2), antibody internalization into tumor and immune cells and the impact of sGD2 on DB and NAXI-mediated ADCC. Results: DB and NAXI mediated a concentration-dependent ADCC response against GD2-positive spheroids and no response against GD2-negative spheroids. DB showed a significantly higher ADCC potency than NAXI in all GD2-positive spheroid models. Binding activity of DB and NAXI was not significantly different. However, the decrease of anti-GD2 antibody binding to viable GD2-positive tumor cells following co-incubation with dead GD2-positive tumor cells or sGD2 was significantly higher for NAXI than DB. Additionally, we found an increased internalization of NAXI compared to DB by tumor cells and particularly CD64+ monocytes. Finally, sGD2 impaired NAXI-mediated ADCC to a significantly greater extent than DB-mediated ADCC. Conclusions: DB has a higher ADCC potency over NAXI at clinically relevant concentrations, attributed to stronger TMDD effects of NAXI compared to DB.

## Simple Summary

Neuroblastoma is a severe childhood cancer, and high-risk patients are treated with antibodies that target the GD2 molecule on tumor cells. This study compares two GD2-specific antibodies—dinutuximab beta, exhibiting moderate GD2-binding affinity, and naxitamab, characterized by higher binding activity—to understand differences in their therapeutic effectiveness. Using three-dimensional tumor models derived from neuroblastoma cells with varying GD2 levels, the study assessed immune responses triggered by these antibodies and examined biological processes that may reduce their activity. Although both antibodies showed similar binding to tumor cells, dinutuximab beta consistently induced stronger immune-mediated killing of GD2-positive tumor cells. This was partly due to naxitamab’s reduced binding when exposed to dead tumor cells or soluble GD2 and its increased internalization by tumor and immune cells. These findings provide important insights into the functional differences between these antibodies and may help improve the clinical use of anti-GD2 immunotherapies.

## 1. Introduction

High-risk neuroblastoma is an aggressive pediatric malignancy of the sympathetic nervous tissue characterized by poor prognosis and few effective therapeutic options. The disialoganglioside GD2 is highly expressed by neuroblastoma cells with limited presence in normal tissues, making it an ideal target for immunotherapy. Monoclonal antibodies (mAbs) targeting GD2 have become an integral part of treatment protocols and significantly contributed to improving survival rates in patients with high-risk neuroblastoma [1].

mAbs have emerged as a cornerstone in cancer therapy and represent one of the fastest-growing classes of biological therapeutics. Their Fc (fragment crystallizable region, Fc) part of the antibody facilitates effector functions such as antibody-dependent cellular cytotoxicity (ADCC) and complement-dependent cytotoxicity (CDC) [2,3]. Additionally, mAbs exhibit favorable biophysical properties such as high conformational stability and solubility, enabling formulation and extended storage with minimal aggregation [4,5].

To date, there are three anti-GD2 mAbs approved for the treatment of high-risk neuroblastoma including dinutuximab (Unituxin^®^), dinutuximab beta (Qarziba^®^, DB), and naxitamab (Danyelza^®^, NAXI). Dinutuximab and DB share the protein sequence of ch14.18, and both are human/mouse chimeric mAbs but differ in the glycosylation pattern. NAXI is a humanized derivative of mAb 3F8. All three anti-GD2 antibodies are of human IgG1 isotype. However, one key difference between NAXI versus DB and dinutuximab is the binding affinity to GD2, with DB showing an intermediate and NAXI exhibiting an approximately 10-fold higher binding affinity to GD2 [2,6].

Recent research indicated that increasing antibody affinity via engineering strategies can enhance ADCC [7]. High affinity also promotes improved antibody internalization, which is particularly beneficial in antibody–drug conjugates [8]. However, other studies showed that high affinity can lead to the bivalent binding of antibodies to their target and therefore reduce the available number of antibodies per antigen [9]. In contrast, moderate-affinity antibodies with faster off-rates may bind monovalently, increasing Fc density on target cells and thereby enhancing immune engagement [9,10]. Moreover, antibodies may be affected by the antigen sink effect, also known as target-mediated drug disposition (TMDD) [11,12,13]. This phenomenon occurs when antibodies bind strongly to abundant target antigens, leading to rapid internalization and degradation of the antibody–antigen complex within cells. As a result, high-affinity antibodies can exhibit nonlinear pharmacokinetics characterized by rapid clearance from the systemic circulation, especially at lower doses [12]. In this context, the role of the affinity difference between DB and NAXI has not been investigated yet.

Moreover, the clinical efficacy of DB and NAXI in patients with high-risk neuroblastoma was reported in separate clinical trials [1,14], and there is no trial so far testing both antibodies in a head-to-head comparison. However, the differences in the patient populations of the reported clinical studies make it difficult to compare the reported outcome data. In this context, we investigated a head-to-head comparison of the intermediate affinity anti-GD2 antibody DB and the higher affinity antibody NAXI using preclinical neuroblastoma spheroid models of varying GD2 expression levels. We determined the capability of both antibodies to mediate cellular and complement-dependent cytotoxicity and show the impact of binding affinity on efficacy and its connection to the TMDD effect.

## 2. Materials and Methods

### 2.1. Ethics Approval and Consent to Participate

Peripheral blood samples were provided by the Department of Transfusion Medicine, University Medicine Greifswald. All experimental protocols involving human participants were reviewed and approved by the Ethics Committee of the Medical Faculty, University Medicine Greifswald, Germany (approval code BB 014/14; approval date 24 January 2014). Written informed consent was obtained from every participant prior to sample collection. All methods were carried out in accordance with the Declaration of Helsinki (1964 and its later amendments) and with the relevant institutional and national regulations and guidelines.

### 2.2. Antibodies

NAXI was purchased from Y-mAbs Therapeutics, Inc. (New York, NY, USA). DB was obtained from EUSA Pharma (Hemel Hempstead, UK) and rituximab from Roche (Basel, Switzerland).

### 2.3. Cell Culture

The human neuroblastoma cells were cultured in respective media under standard cell culture conditions (37 °C/5% CO_2_). LAN-1 cells were cultured in RPMI (PAN BIOTECH, Aidenbach, Germany, P04–16520) supplemented with 10% FCS and 100 U/mL penicillin and 0.1 mg/mL streptomycin (1× P/S; PAN BIOTECH, P06–07100). CHLA-20, COG-N-440, and COG-N-519 cells were cultured in IMDM (PAN BIOTECH, P04–20250) supplemented with 4 mM stable glutamine, 20% FCS, 1× Insulin–Transferrin–Selenium (ITS) solution I (PAN-BIOTECH; P07-03110) and 1× P/S. SHEP-2, SK-N-SH, and COG-N-519 cells were cultured using MEM Eagle (PAN BIOTECH, P04-08056) supplemented with 10% FCS and 1× P/S. To detect potential mycoplasma contamination, all cell lines were regularly analyzed using a MYCOALERT detection Kit (Lonza Cologne GmbH, Cologne, Germany, LT07–318); only mycoplasma-negative cells were utilized for experiments. For ADCC experiments, human peripheral blood mononuclear cells (PBMCs) were separated from whole blood concentrates without serum of healthy donors using the Pancoll separating method (human, density 1.077 g/mL, BIOTECH, P04-60500) and cultured for 72 h in RPMI supplemented with 10% FCS, 50 µM, 100 IU/mL IL-2, β-mercaptoethanol, and 1× P/S. LAN-1 and SK-N-SH were commercially obtained from the American Type Culture Collection (ATCC, Manassas, VA, USA) and the other cell lines were provided by the childhood cancer repository (Texas Tech University Health Sciences Center, Lubbock, TX, USA).

### 2.4. Lentiviral Transduction

For the generation of neuroblastoma tumor cells expressing near-infrared reporter protein (iRFP-680) used as a viability marker, a second-generation lentiviral vector system was utilized. To produce recombinant lentivirus, CalPhos Mammalian Transfection Kit (Takara Bio Europe, Saint-Germain-en-Laye, France) was used according to the manufacturer’s protocol for co-transfection of Lenti-X™ 293T cells with purified pVSV-G envelope expressing plasmid (Addgene, Watertown, MA, USA), psPAX2 (Addgene, USA) vector encoding virus polymerase and packaging genes, and lentiviral vector pWPXL (Addgene, USA) encoding for iRFP-680. Tumor cells (target cells) were transduced using 8 mg/mL Polybrene (Merck, Burlington, MA, USA) and virus-containing supernatants. After 24 h, medium was replaced by a fresh standard culture medium, and after 72 h, target cells were checked for iRFP-680 fluorescence using the IncuCyte^®^ SX5 live-cell analysis system (Sartorius, Göttingen, Germany).

### 2.5. Long-Term Live-Cell Spheroid Viability Assay

Tumor spheroids were established by seeding 3000 iRFP-680-expressing tumor cells per well into a 384 ultra-low attachment plate (S-BIO, Hudson, NH, USA, PrimeSurface^®^, MS-90384UZ), centrifuged at 150× *g* for 10 min and cultured under cell culture conditions. After 72 h, tumor spheroids were deployed for experiments using the IncuCyte^®^ SX5 live-cell analysis system (Sartorius, Germany) with image acquisitions every 8 h. Viability was determined as the ratio of integrated spheroid fluorescence intensity of every time point and fluorescence at baseline (0 h). The area under the curve for each time period was calculated using the following formula: ((viability-time point 1 (t1) (%) + viability t2)/2) × (t2 − t1). Experiments were performed in six technical replicates, and viability is given in % ± SEM.

### 2.6. ADCC and CDC Live-Cell Viability Assay

To determine antibody-dependent cytotoxicity against neuroblastoma spheroids in a long-term live-cell spheroid viability assay, 75.000 effector cells (PBMCs) per well, as well as respective therapeutic antibody in a concentration range of 10^−5^–1 µg/mL, were added to the tumor spheroids (ADCC) and cultured in a total volume of 200 µL for 168 h (7 days) under cell culture conditions. PBMCs were taken from at least five different donors to ensure accuracy and reliability of the results.

In complement-dependent cytotoxicity experiments, 12.5% active human serum was used instead of PBMCs. To assess reproducibility and account for donor-specific variability in complement activity, independent experiments were performed using sera from multiple donors.

Tumor spheroids were only treated with PBMCs alone (antibody-independent cellular cytotoxicity, AICC) or heat-inactivated serum alone without therapeutic antibodies, and untreated tumor spheroids (medium) served as controls. To determine the impact of soluble target antigen, we added 100 nM and 1000 nM sGD2 (Merck, Burlington, MA, USA, 345743-500UG) in addition to the ADCC setting.

### 2.7. CDC Calcein–AM Assay

CDC was assessed using a calcein–acetoxymethyl ester (AM)-based cytotoxicity assay as published [15]. In brief, 5000 calcein–AM-labeled, GD2-positive neuroblastoma cells were incubated with patient serum at a final concentration of 12.5% for 4 h. Heat-inactivated serum alone (i.e., without leukocytes) served as the negative control for CDC. After the incubation period, cytotoxicity was quantified by measuring the calcein fluorescence in the supernatant at excitation and emission wavelengths of 495 nm and 515 nm, respectively.

### 2.8. Analysis of GD2 Expression by Neuroblastoma Cells by Flow Cytometry

To determine GD2 expression by flow cytometry, iRFP-680-expressing neuroblastoma cells were harvested at 80–100% confluency. A total of 1 × 10^6^ cells per sample were washed in washing buffer (1× PBS without Ca and Mg (CAPRICORN SCIENTIFIC, Ebsdorfergrund, Germany, PBS-1A) supplemented with 2% BSA and 1 mM EDTA; 300× *g*, 5 min) and incubated with PE-conjugated anti-GD2 antibody (BioLegend, San Diego, CA, USA, #357304, mouse IgG2a, κ, clone 14G2a, 1:80) for 20 min at 4 °C in the dark. After removing unbound antibodies by centrifugation (300× *g*, 5 min), cells were resuspended in washing buffer and analyzed using a BD CANTO II flow cytometer and FACS Diva software V10.10.0 (BD Biosciences, Heidelberg, Germany). A total of 20,000 live cells were analyzed by default. Samples stained with PE-labeled mouse IgG2a, κ isotype control antibody (BioLegend, #400214) were used to detect unspecific binding; unstained samples served as control.

### 2.9. Assessing Loss of Anti-GD2 Antibody Binding Activity by Pre-Incubation with GD2-Positive Dead Neuroblastoma Cells Using Flow Cytometry

To compare the off-rate characteristics of NAXI and DB, we exposed both antibodies to GD2-positive dead neuroblastoma cells before staining viable LAN-1 or CHLA-20 neuroblastoma cells. For this, neuroblastoma cells were cultured until 80–100% confluency, harvested, and washed using washing buffer (300× *g*, 5 min). To kill the neuroblastoma cells, 0.5 × 10^6^/mL cells were exposed to 65 °C for 5 min followed by 4 °C for 5 min (dead cells were identified with DAPI staining). Dead cells were then incubated with either NAXI or DB (10–0.001 µg/mL) for 60 min at 4 °C in a reaction volume of 1000 µL. After centrifugation (300× *g*, 5 min), the supernatants were used for the staining of live cells (0.5 × 10^6^, 20 min at 4 °C) (samples 1). In parallel, 0.5 × 10^6^ live cells were stained with NAXI or DB (10–0.001 µg/mL) for 20 min at 4 °C (sample 2). After washing cells (washing buffer, 300× *g*, 5 min), the secondary antibody was incubated (Alexa Fluor 488-conjugated anti-human IgG1, BioLegend, #410706, rat IgG2a, κ, clone M1310G05, application 1:20 in 100 µL incubation volume, 20 min, 4 °C). Cells were washed again (washing buffer, 300× *g*, 5 min) and resuspended in washing buffer for the detection of the mean fluorescence intensity (MFI) of GD2 by flow cytometry, as described above. Tumor cells stained with the secondary antibody only, as well as unstained cells, served as negative controls. The loss of fluorescence intensity is given as fold decrease calculated as the ratio of the gMFI GD2 of live cells (sample 2) and live cells stained with supernatant obtained following pre-incubation with dead cells (sample 1). Experiments were performed in at least four biological replicates; values are given in fold change ± SEM.

### 2.10. Assessing Loss of Anti-GD2 Antibody Binding Activity in Presence of Soluble GD2 Using Flow Cytometry

To assess the off-rate characteristics of NAXI and DB in presence of sGD2, NAXI and DB were used in a competitive binding assay to CHLA-20 tumor cells in addition of 10, 100, and 1000 nM sGD2. For this, NAXI or DB was diluted in washing buffer in a concentration range from 0.001 to 1 µg/mL. GD2 (Merck, CAS# 65988-71-8) was diluted in methanol (Carl Roth GmbH + Co. KG, Karlsruhe, Germany, # 4627.5) at 10, 100, and 1000 nM. First, 0.001–1 µg/mL antibody and 10, 100, and 1000 nM GD2 were incubated for 30 min in a reaction volume of 1000 µL. Antibody solution added with methanol only (without GD2) as well as washing buffer, and methanol (without antibody and GD2) served as control. Second, CHLA-20 neuroblastoma cells were harvested at a confluency of 80–100% and washed using washing buffer (300× *g*, 5 min). A total of 0.5 × 10^6^ live neuroblastoma cells resuspended in 50 µL of washing buffer were added to the GD2 antibody solution and controls. After incubation for 20 min at 4 °C, samples were centrifuged (300× *g*, 5 min), and the supernatant was removed, followed by another washing step (washing buffer, 300× *g*, 5 min). Then, samples were incubated with the secondary antibody (Alexa Fluor 488-conjugated anti-human IgG1, BioLegend, #410706, rat IgG2a, κ, clone M1310G05, application 1:20 in 100 µL incubation volume, 20 min, 4 °C). After washing (washing buffer, 300× *g*, 5 min), cells were resuspended in washing buffer for cytometric analysis of GD2 gMFI, as described above. Tumor cells stained with the secondary antibody only, as well as unstained cells, served as negative controls. The loss of fluorescence intensity is given as fold decrease calculated as the ratio of the gMFI GD2 of live cells and live cells stained with GD2 antibody solution. Experiments were performed in at least four biological replicates; values are given in fold change ± SEM.

### 2.11. Antibody-Internalization Assay Using Live-Cell Imaging

To measure antibody internalization in relation to binding affinity and concentration of the therapeutic antibodies, we established an internalization assay using NAXI or DB tagged with a pH-sensitive dye and the live-cell imaging method (IncuCyte^®^). First, a desalting and buffer exchange procedure of both antibodies was performed to remove low molecular weight components and primary amines, which may inhibit protein labeling. For this, Zeba Spin Desalting Columns (ThermoFisher Scientific, Waltham, MA, USA, #89889) were used according to the manufacturer’s instructions. Secondly, antibody labeling was conducted using a pHrodo™ iFL Red Antibody Labeling Kit (ThermoFisher Scientific, # P36021) followed by the measurement of protein contraction and degree of labeling (DOL) according to the manufacturer’s protocol to obtain a covalently attached fluorogenic pH probe. DOL was calculated according to the following formula:

$$DOL=\frac{Abs_{560}\;\times \;eProtein}{eLabel\times [Abs_{280}-Correction\;factor_{280}\;\times \;Abs_{560}]}$$

The absorbance was measured using a ThermoScientific Varioskan™ LUX plate reader at 280 nm for protein concentration and 560 nm for the label dye as per the manufacturer’s instructions. The degree of labeling of DB and NAXI was similar with 2.27 and 2.23, respectively, rendering corrections for DOL unnecessary (correction factor = 1.02).

For the antibody internalization assay, 10,000 tumor cells or 100,000 PBMCs per well were seeded into a 96-well plate (Corning, New York, NY, USA, 3595) and cultured for 24 h. Then, pHrodo™ iFL Red-labeled NAXI or DB in a concentration from 1 to 0.001 µg/mL diluted in standard culture medium were added to the wells. A total of 1 µg/mL rituximab and non-treated cells served as controls. Antibody internalization was assessed using the IncuCyte^®^ SX5 live-cell analysis system with image acquisitions every 2 h. Antibody internalization was calculated as a ratio of integrated fluorescence intensity of every time point and fluorescence at baseline (0 h). Values are shown as areas under the total integrated red fluorescence intensity curve ± SEM.

### 2.12. Measurement of Antibody Internalization by Leukocytes Using Flow Cytometry

To measure antibody internalization by leukocytes, 100 µL of fresh whole blood collected in heparin tubes were incubated with 1 µg of pHrodo-labeled NAXI or DB (detection equivalent to PE) for 4 h under cell culture conditions. For immunostaining, 20 µL of anti-human FcR blocking reagent (Miltenyi Biotec, Bergisch Gladbach, Germany, #130-059-901) was added per sample and incubated for 10 min at 4 °C, followed by adding of APC/Vio770-labeled anti-human CD45 antibody (Miltenyi Biotec #130-110-635, human IgG1, REA 747, 1:50) and APC-conjugated anti-human CD64 antibody (BioLegend #399510, mouse IgG1κ, clone S18012C, 1:40). After incubation for 30 min at 4 °C in the dark, samples were washed (4 mL of washing buffer) and centrifuged at 300× *g* for 5 min, and the supernatant was aspirated. To remove red blood cells, 2 mL of erythrocyte lysis buffer was added, gently mixed, and incubated for 10 min at room temperature. After adding 2 mL of washing buffer, samples were again centrifuged (300× *g* for 5 min), and the supernatant was discarded. For flow cytometry measurement, the cell pellet was resuspended using 500 µL of washing buffer. A total of 0.1 µg/mL 4′,6-diamidino-2-phenylindole (DAPI) was added 5 min before acquisition to exclude dead cells from analysis. Flow cytometry analysis was performed as described above.

To investigate the expression of GD2 by leukocytes, whole blood samples without addition of pHrodo-labeled antibody were stained with anti-human CD45-APC/Vio770, anti-human CD64-APC (see above), and PE-conjugated anti-GD2 antibody (BioLegend, #357304, mouse IgG2aκ, clone 14G2a, 1:80) in the manner described above.

### 2.13. Statistics

Statistical analyses were conducted using GraphPad version 10.4.1. Normality was assessed before further analysis. For comparisons between two groups, a two-sided *t*-test was used, while one-way ANOVA was used for comparisons involving more than two groups, followed by the appropriate post hoc test. Data are presented as mean ± SEM or as fold change compared to untreated control ± SEM.

## 3. Results

### 3.1. DB- Versus NAXI-Mediated Cellular Cytotoxicity

Antibody affinity to its target might affect its effector function [13]. Therefore, we first investigated the capability of intermediate affinity DB and higher affinity NAXI to mediate ADCC against neuroblastoma spheroids using peripheral blood mononuclear cells (PBMCs) from healthy donors in a long-term life-cell viability assay over seven days, as described previously [16]. As a target, we utilized spheroids derived from six different neuroblastoma cells ranging from no to high GD2 expression (Supplemental Figure S1A). Clinically relevant antibody concentrations ranging from 10^−5^ to 1 µg/mL were tested. All were genetically modified to express near-infrared fluorescent protein (iRFP-680), which is used as a viability marker (Supplemental Figure S1B,C). Spheroids treated with PBMCs only served as negative controls (antibody-independent cellular cytotoxicity, AICC).

We observed no ADCC activity of DB or NAXI against neuroblastoma cells with low or no GD2 expression (SHEP-2, SK-N-SH, COG-N-519) at any concentration, suggesting that GD2 surface expression is crucial for antibody-mediated Fcγ receptor (FcγR) activation (Supplemental Figure S2).

**Figure 1:**
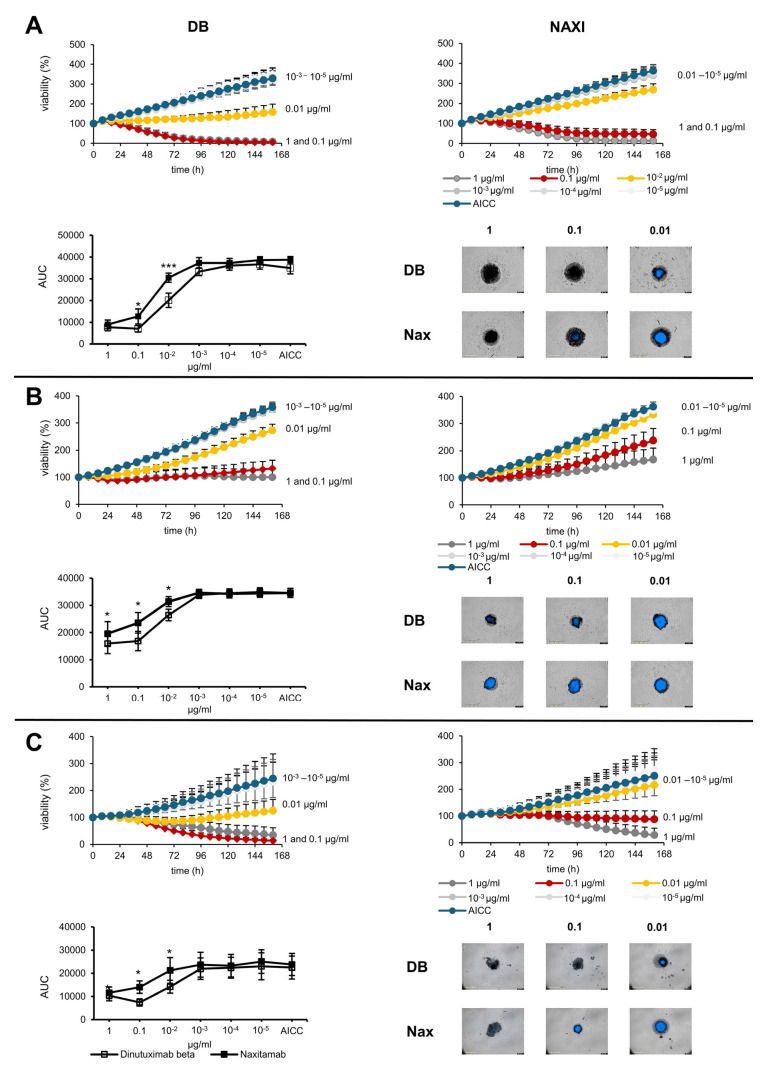
Comparison of antibody-dependent cellular cytotoxicity mediated by antibodies with intermediate (DB) and higher affinity (NAXI) to GD2 against spheroids of LAN-1 (**A**), CHLA-20 (**B**), and COG-440 (**C**) cells. The viability of spheroids was determined by monitoring near-infrared fluorescent protein (iRFP680) expression by tumor cells. Tumor spheroids were treated with a concentration series of the GD2-specific antibodies dinutuximab beta (DB, intermediate affinity) and naxitamab (NAXI, higher affinity) co-incubated with 75,000 PBMCs for 168 h. Spheroids treated with PBMCs only (without antibodies) served as negative controls (antibody-independent cellular cytotoxicity, AICC, blue curves). Viability was calculated as the total integrated spheroid fluorescence of the respective time point divided by the total fluorescence at time point 0 h. Graphs show the viability of spheroids at antibody concentrations of 10^−5^–1 µg/mL and AICC, as indicated in the legend. The area under the curve (AUC) was calculated for each viability curve and displayed in the lower left panels. Representative images show spheroids after 160 h of treatment with 1, 0.1, and 0.01 µg/mL of DB or NAXI, 4× magnification. Data are shown as means from at least five independent experiments (done in four replicates) ± SEM. Paired *t*-test: *** *p* < 0.001, * *p* < 0.05 DB vs. NAXI area under the viability curve.

In GD2 high-expressing tumor cells (i.e., LAN-1, CHLA-20, and COG-440), we found that both antibodies were immunologically active in the concentration range of 1–0.01 µg/mL (Figure 1A–C). However, in the range of 0.1–0.01 µg/mL, DB induced a significantly higher ADCC response against the GD2 high-expressing neuroblastoma spheroids (LAN-1, CHLA-20, COG-N-440, Figure 1A–C) compared to NAXI. DB also mediated a superior anti-tumor activity at 1 µg/mL against CHLA-20 cells compared to NAXI. Importantly, DB was still active at very low concentrations of 0.01 µg/mL, whereas NAXI showed no significant ADCC response compared to control (AICC).

These data indicate a higher ADCC potency of DB compared to NAXI, particularly at lower antibody concentrations.

### 3.2. Complement Activity Mediated by DB and NAXI

**Figure 2:**
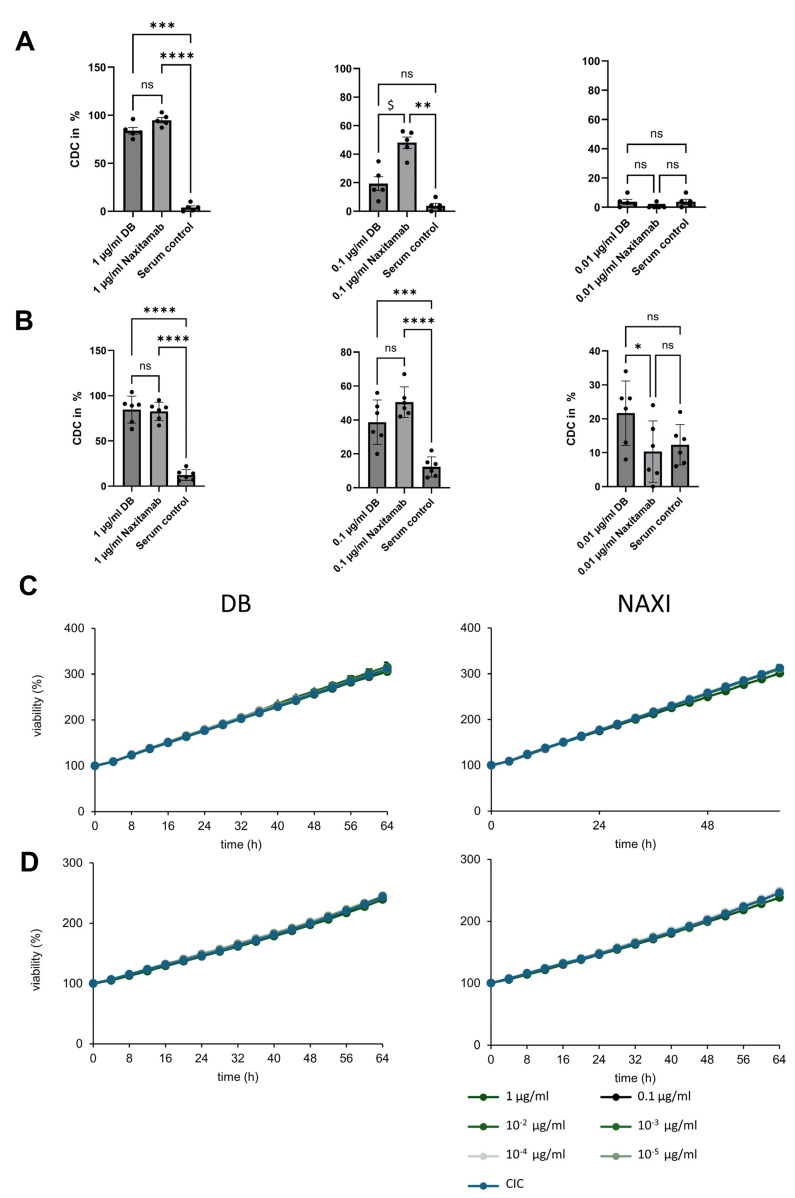
Comparison of complement-dependent cytotoxicity mediated by the anti-GD2 antibodies dinutuximab beta (DB, intermediate affinity) and naxitamab (NAXI, higher affinity) in a two-dimensional (**A**,**B**) and three-dimensional spheroid model (**C**,**D**). (**A**) LAN-1 and (**B**) CHLA-20 neuroblastoma cells (2500 cells/well) were stained with calcein–AM and incubated with 12.5% active human serum in the presence of a concentration series of DB or NAXI (0.01–1 µg/ml) or absence of antibodies serving as control (complement-independent cytotoxicity, CIC). Cytotoxicity was assessed after 4 h by measuring calcein–AM release. (**C**,**D**) For the spheroid assay, 10,000 LAN-1 (**C**) or CHLA-20 (**D**) cells stably expressing a near-infrared fluorescence marker were used for seed spheroid formation. Spheroids were treated with 12.5% active human serum and a concentration series of DB or NAXI, with viability determined as the total integrated spheroid fluorescence of the respective time point divided by the total fluorescence at time point 0 h. Statistical differences were analyzed using ANOVA, comparing all groups and using the Holm–Šídák method. Statistical significance is indicated as follows: * *p* < 0.05, ** *p* < 0.01, *** *p* < 0.001, **** *p* < 0.0001 versus untreated control; ^$^ *p* < 0.01 versus DB; ns = not significant.

We assessed the role of antibody affinity to GD2 for CDC activity mediated by DB and NAXI against CHLA-20 and LAN-1 neuroblastoma cells using a two-dimensional (2D) calcein–AM assay where cells are scattered on a 2D surface, as previously described [15], and compared results to the three-dimensional (3D) spheroid long-term live-cell imaging assay [16]. Human active serum (blood group AB) at 12.5% final concentration was used as a source for complement. In the 2D model, complete lysis of tumor cells by CDC was observed at antibody concentrations of 1 µg/mL for DB and NAXI, and analysis substantially decreased at 0.1 and 0.01 µg/mL (Figure 2A,B). At 0.1 µg/mL, NAXI induced a stronger CDC response against GD2 high-expressing LAN-1 cells, whereas the effects of NAXI and DB were comparable against CHLA-20 cells. In line with the ADCC results, DB—but not NAXI—mediated CDC at 0.01 µg/mL against CHLA-20 cells (Figure 2B).

Most importantly, when CDC was investigated against 3D spheroids in a long-term assay over seven days, LAN-1 and CHLA-20 cells were completely resistant to CDC mediated by both antibodies (Figure 2C and Figure 2D, respectively).

Our data suggest that CDC requires higher antibody concentrations than ADCC for tumor cell lysis and that spheroids are resistant to CDC. This underscores that ADCC is the main mode of action for both antibodies.

### 3.3. Comparison of Binding Affinity of DB Versus NAXI

Studies showed that high-affinity antibodies have disadvantages over naturally occurring intermediate-affinity antibodies due to differences in binding valencies [9]. Antigen binding by high-affinity mAbs is preferentially bivalent compared to monovalent binding by intermediate-affinity mAbs. Therefore, antigen coverage by high-affinity mAbs may be reduced compared to intermediate-affinity mAbs. To test this, we incubated the high GD2-expressing neuroblastoma cells CHLA-20 and LAN-1 with DB or NAXI in a ten-fold concentration series, followed by staining with a human IgG1-specific secondary antibody and flow cytometric analysis.

**Figure 3:**
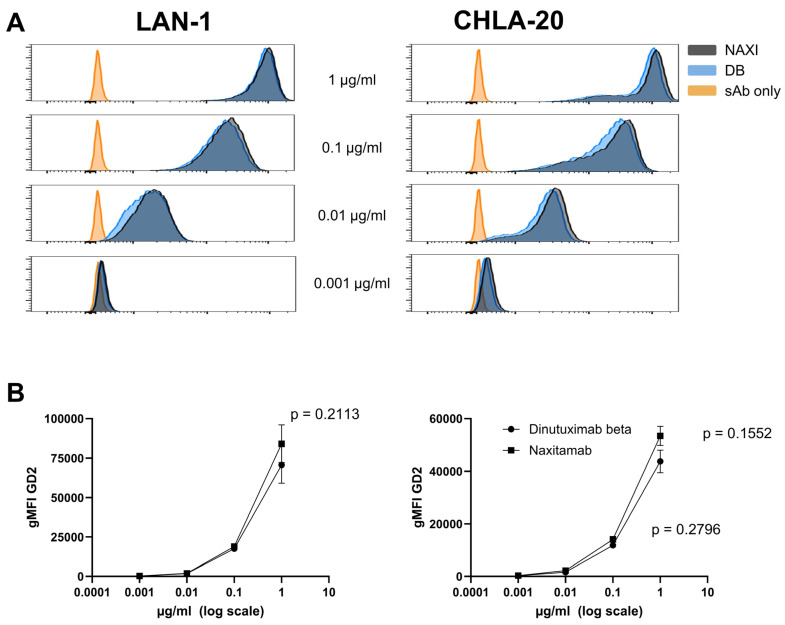
Comparison of intermediate (dinutuximab beta, DB) and higher affinity (naxitamab, NAXI) anti-GD2 antibody binding to neuroblastoma cells LAN-1 and CHLA-20. Tumor cells (1 × 10^6^) were incubated with varying concentrations (0.001–1 µg/mL) of DB or NAXI, followed by staining with an FITC-conjugated secondary antibody. (**A**) Representative histograms of GD2 staining with DB (blue) or NAXI (black) of neuroblastoma cells LAN-1 (**left** panel) and CHLA-20 (**right** panel) are shown. Staining with the secondary antibody alone served as negative control (yellow). (**B**) Graphs depicting mean GD2-specific geometric mean fluorescence intensity (gMFI) for NAXI (squares) and DB (circles) with LAN-1 (**left** panel) and CHLA-20 (**right** panel) cells. Results are presented as the mean of four (LAN-1) to seven (CHLA-20) replicates with error bars representing SEM. Statistical difference was calculated using *t*-test.

Across all concentrations, we detected a similar mean fluorescence intensity for NAXI and DB binding to LAN-1 and CHLA-20 cells, with slightly higher signals for NAXI at higher concentrations. However, this difference was not statistically significant (Figure 3A,B).

In conclusion, high-affinity mAb NAXI exhibited similar binding activity to GD2-expressing neuroblastoma cells comparable to intermediate-affinity mAb DB, suggesting that the antigen coverage model as described above does not apply to NAXI versus DB.

### 3.4. Role of On-Target Off-Viable Tumor Binding

Since we did not observe a difference in opsonization of GD2-expressing tumor cells between anti-GD2 mAbs NAXI versus DB, we further investigated whether TMDD may impact antibody concentration. To explore the off-rate characteristic of intermediate DB and higher affinity NAXI, we pre-incubated the antibodies (10^−3^ to 1 µg/mL) with heat-killed tumor cells (LAN-1 and CHLA-20). After incubation, we collected supernatants containing unbound antibodies to stain viable tumor cells. Direct staining of viable cells (without pre-incubation with dead cells) served as control. Flow cytometry was used to determine the binding of the remaining antibodies to live cells by measuring the geometric mean fluorescence intensity (gMFI) as a parameter for the remaining functional antibody amount.

**Figure 4:**
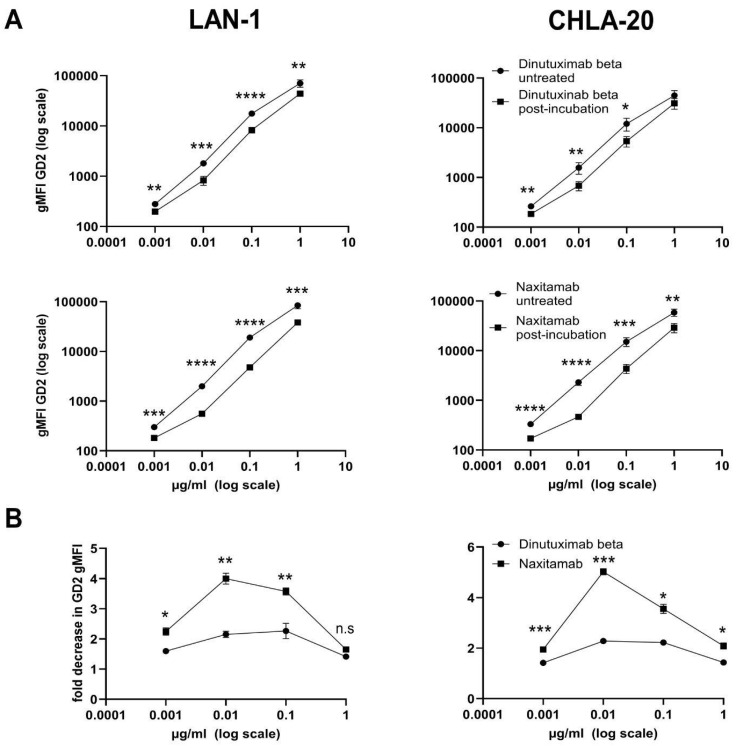
Loss of GD2-binding activity of dinutuximab beta (DB) and naxitamab (NAXI) following pre-incubation with dead GD2-expressing tumor cells. Antibodies (1–0.001 µg/mL) were pre-incubated with LAN-1 (**left** panel) and CHLA-20 (**right** panel) cells that were heat-killed (treatment at 56 °C followed by incubation on ice). After incubation, the cell-antibody suspension was centrifuged, and supernatants were used for the staining of viable LAN-1 and CHLA-20 cells. (**A**) Binding activity, expressed as gMFI GD2 of DB (**top** panels) and NAXI (**bottom** panels) to viable neuroblastoma cells (LAN-1, **left** panel; CHLA-20 **right** panel) with (squares) and without (circles) pre-incubation with dead LAN-1 (**left** panel) and dead CHLA-20 (**right** panel) cells. (**B**) Fold decrease in binding activity (gMFI) of DB (circles) and NAXI (squares) for LAN-1 (**left** panel) and CHLA-20 (**right** panel) cells. Statistical comparisons in (**A**) assess with and without pre-incubation binding activity for DB and NAXI, while (**B**) analyze the loss of gMFI of NAXI versus DB. (**A**) ANOVA with the appropriate post hoc test and (**B**) two-sided, unpaired *t*-tests to compare respective concentrations were used for statistical analysis, with significance indicated as follows: * *p* < 0.05, ** *p* < 0.01, *** *p* < 0.001 and **** *p* < 0.0001 versus DB or NAXI; n.s = not significant.

For both mAbs, DB and NAXI, the detected amount of mAB after incubation with dead tumor cells was significantly reduced over the entire concentration range (10^−3^–1 µg/mL) compared to controls (Figure 4A). Importantly, the GD2-binding capacity of NAXI decreased by up to 5-fold for both cell lines, whereas the capacity of DB decreased by only 2-fold (Figure 4B). The largest decrease in GD2-gMFI occurred at 0.01 and 0.1 µg/mL for NAXI in both cell lines compared to DB. In LAN-1 cells the loss of GD2-gMFI was not significant at 1 µg/mL, but at 0.1 µg/mL–0.001 µg/mL, whereas in CHLA-20 even at 1 µg/mL a significantly higher loss of GD2-gMFI for NAXI compared to DB was detected (Figure 4B).

In conclusion, we could identify a GD2-dependent antibody-disposition effect mediated by dead tumor cells that affects NAXI to a larger extent compared to DB, which may explain the observed differences in the ADCC response (Figure 1).

### 3.5. Effect of Soluble GD2 on the Binding of NAXI and DB to Neuroblastoma Cells

GD2 is a surface molecule that is shedded into the blood circulation [17]. Therefore, we investigated the impact of soluble GD2 (sGD) on the binding of NAXI and DB to GD2 high-expressing neuroblastoma cells CHLA-20 at antibody concentrations ranging from 10^−4^ to 1 µg/mL in the presence of sGD2 using flow cytometry. The gMFI was used as a parameter for binding intensity. The concentration of sGD2 to be added to the binding experiments was selected based on reported median and maximum concentration levels from patients with high-risk neuroblastoma (100 nM and 1000 nM, respectively) also including the level observed in healthy controls (10 nM) [17].

**Figure 5:**
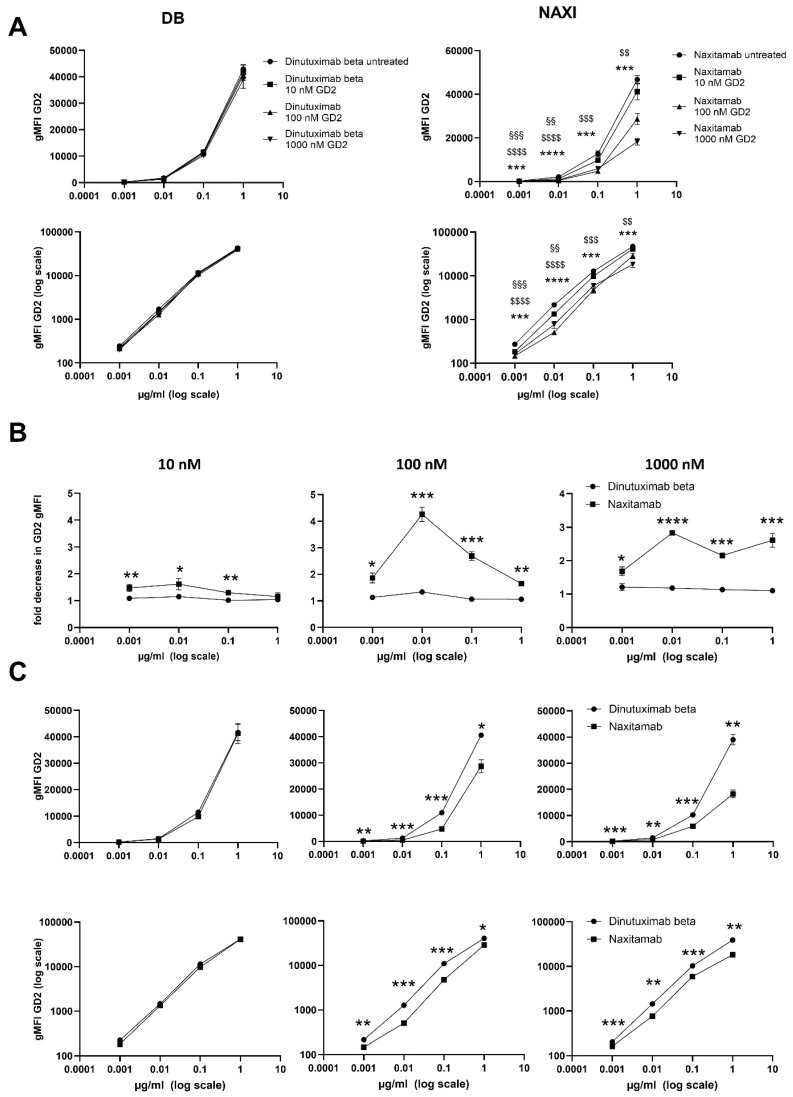
Reduction of anti-GD2 antibody binding to neuroblastoma cells by soluble GD2 (sGD2). NAXI and DB (0.001–1 µg/mL) were used in a competitive binding assay to GD2 high-expressing CHLA-20 tumor cells in the presence of 10, 100, and 1000 nM sGD2. An FITC-conjugated human IgG1-specific secondary antibody was used for the detection of bound anti-GD2 mAbs. (**A**) DB (**left** panels) and NAXI (**right** panels) show binding (gMFI GD2) in the presence and absence of sGD2 (10–1000 nM) with linear- (**upper** panels) and log-y-scales (**lower** panels). (**B**) Fold decrease in the binding of anti-GD2 antibodies to neuroblastoma cells (gMFI GD2) in the presence of sGD2 (NAXI: squares; DB: circles). (**C**) Direct comparison of DB (circles) and NAXI (squares) binding to GD2-expressing neuroblastoma cells incubated with 10 nM (**left** panel), 100 nM (**center** panel), and 1000 nM (**right** panel) sGD2. Results are shown using linear- (**upper** panels) and log-scales (**lower** panels). Data represent the mean of gMFI ± SEM from four independent experiments. Statistical comparisons (**A**) One-way ANOVA 1000 nM (*), 100 nM ($), and 10 nM (§) vs. 0.001–1 µg/mL. $$/§§ *p* < 0.01, ***/$$$/§§§ *p* < 0.001, and ****/$$$$ *p* < 0.0001 versus untreated NAXI. (**B**,**C**) Two-sided *t*-tests were used for statistical analysis, with significance indicated as follows: * *p* < 0.05, ** *p* < 0.01, *** *p* < 0.001 and **** *p* < 0.0001 versus NAXI.

We observed no significant adverse effect of sGD2 on the binding of DB to neuroblastoma cells at any tested antibody or sGD2 concentration (Figure 5A, left upper and lower panels, log scale). This finding was in contrast to the binding of NAXI, which was significantly reduced at all applied antibody concentrations when co-incubated with 100 nM and 1000 nM sGD2. Using 10 nM sGD2, a significant reduction of binding of NAXI to neuroblastoma cells was observed at lower antibody concentrations (Figure 5A, right upper and lower panels).

We calculated the fold decrease of antibody binding to neuroblastoma cells in the presence of sGD2 by dividing the gMFI intensity determined in the absence of sGD2 by the intensity with sGD2. The fold decrease by the addition of 100 nM and 1000 nM sGD2 was significantly higher for NAXI in contrast to DB across all antibody concentrations (Figure 5B). With 10 nM sGD2, the decrease of NAXI binding was significant at mAb concentrations ranging from 0.001 to 0.1 µg/mL with no effect on DB binding, indicating that low sGD2 levels also impact NAXI binding to GD2-expressing neuroblastoma cells.

We also compared DB and NAXI side by side. The binding curves for both antibodies overlapped in the presence of 10 nM sGD2, a concentration level below what is typically observed in neuroblastoma patients (Figure 5C, left panel). However, in the presence of 100 nM (Figure 5C, middle panel) and 1000 nM (Figure 5C, right panel) sGD2, the binding of NAXI to neuroblastoma cells was significantly reduced at all antibody concentrations compared to DB (Figure 5C).

These findings demonstrate that NAXI, but not DB, is affected by a significant sink effect attributed to the presence of sGD2 at clinically relevant concentrations.

### 3.6. Internalization of Anti-GD2 Antibodies by Neuroblastoma Cells

To investigate the internalization of NAXI and DB by neuroblastoma cells, both mAbs were labeled with a pH-sensitive fluorochrome, pHrodo, ensuring the same degree of labeling as described in the methods section.

**Figure 6:**
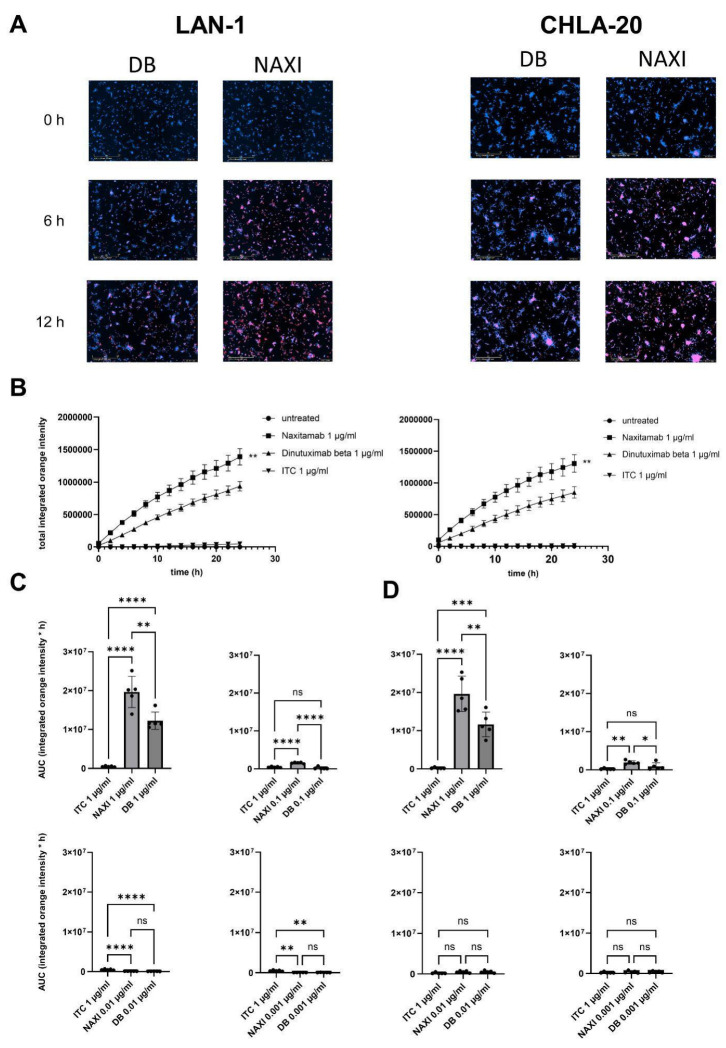
Internalization of dinutuximab beta (DB) and naxitamab (NAXI) by tumor cells. (**A**) Representative images of LAN-1 (**left** panel) and CHLA-20 (**right** panel) after 0, 6, and 12 h of treatment with pH-sensitive pHrodo-labeled DB (pHrodo-DB) and NAXI (pHrodo-NAXI). Internalization by viable cells is indicated by purple fluorescence, 10× magnification. (**B**) Quantification of anti-GD2 antibody internalization over time for LAN-1 (**left** panel) and CHLA-20 (**right** panel). Area under the internalization curve (AUC; integrated orange fluorescence intensity over time) for LAN-1 (**C**) and CHLA-20 (**D**) treated with pHrodo-DB and pHrodo-NAXI. Tumor cells were stably transduced to express near-infrared fluorescence (NIR), which served as a viability marker, as previously described [16]. A total of 10,000 tumor cells were incubated with pHrodo-labeled DB or NAXI at concentrations ranging from 0.001 to 1 µg/mL. pHrodo–rituximab was included as an IgG1 isotype control. Data represent the mean ± SEM from five independent experiments, each performed in quadruplicate. Statistical comparisons of the AUC (orange fluorescence) versus between NAXI and DB (*) and control were performed using ANOVA followed by appropriate post hoc tests. Statistical significance is indicated as follows: **** *p* < 0.0001, *** *p* < 0.001, ** *p* < 0.01, * *p* < 0.05 versus isotype control; ns = not significant.

NAXI and DB showed a rapid internalization within 24 h (Figure 6A,B) compared to the human IgG1 isotype control (rituximab). The internalization rate of NAXI was significantly higher at 0.1 and 1 µg/mL in high GD2-expressing LAN-1 and CHLA-20 neuroblastoma cells compared to DB (Figure 6C,D). At concentrations below 0.1 µg/mL, no significant level of internalization was detectable (Figure 6C,D).

In summary, NAXI was internalized into tumor cells at a higher rate and to a higher degree than DB.

### 3.7. Internalization of mAbs by Leukocytes

We assessed antibody internalization by immune cells using a live-cell assay with pH-sensitive pHrodo-labeled NAXI and DB (1 µg/mL). Results were confirmed with flow cytometry. For that, leukocyte subpopulations were identified via standard side scatter/CD45 gating to distinguish between lymphocytes, monocytes, and granulocytes. In addition, CD64 and GD2 expression was measured in each subpopulation to determine whether antibody internalization correlates with these receptors.

**Figure 7:**
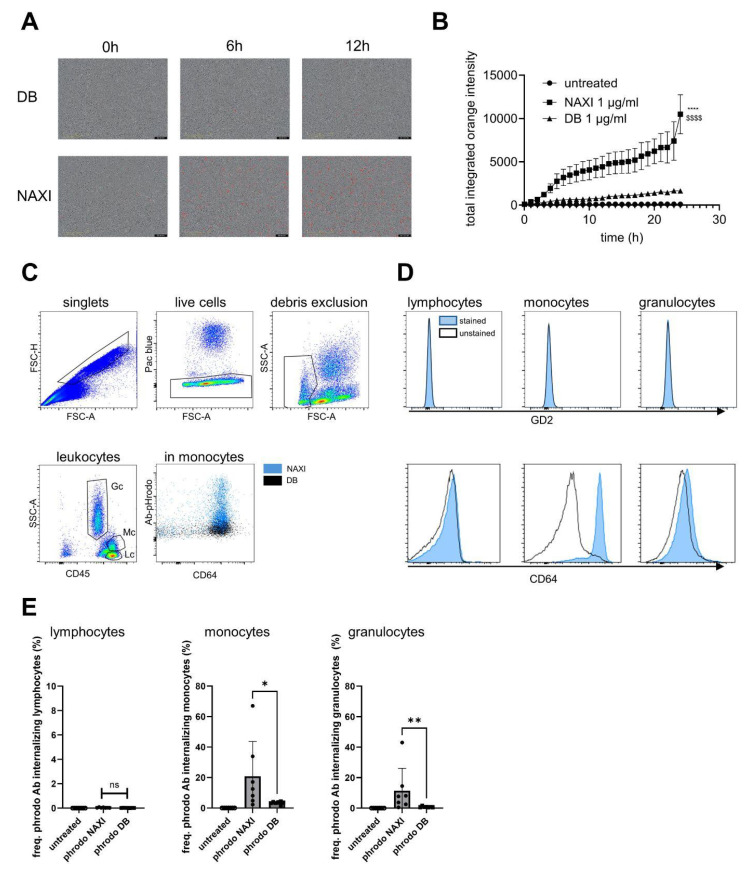
Dinutuximab beta and naxitamab internalization by immune cells. A total of 10,000 leukocytes were incubated with 1 µg/mL pHrodo-DB or pHrodo-NAXI; 10× magnification. (**A**) Representative images of immune cells after 0, 6, and 12 h of treatment with pH-sensitive pHrodo-labeled DB (pHrodo-DB, **upper** panel) and NAXI (pHrodo-NAXI, **lower** panel). Internalization by leukocytes is indicated by red fluorescence. (**B**) Internalization of NAXI (squares) and DB (triangles) and no antibodies (control, circles) over time. (**C**) Gating strategy to identify leukocyte subpopulations using forward and side scatter and CD45. Representative Dot Plot of monocytes after internalization of pHrodo-labeled NAXI or DB and CD64 staining. (**D**) GD2 (**upper** panel) and CD64 (**lower** panel) expression of lymphocytes (**left**), monocytes (**center**), and granulocytes (**right**). (**E**) Frequency of lymphocytes (**left**), granulocytes (**center**), and monocytes (**right**), which internalized pHrodo-NAXI (closed circles) and DB (squares) and control without antibodies (open circles). Data represent the mean ± SEM from at least four independent experiments. (**B**) Statistical comparisons of the AUC (orange fluorescence) versus control (without addition of antibodies) and DB ($) were performed using ANOVA followed by appropriate post hoc tests. Statistical significance is indicated as follows: **** *p* < 0.0001 vs. DB; $$$$ *p* < 0.0001 vs. untreated control. (**E**) Paired *t*-test. * *p* < 0.05 vs. DB (monocytes), ** *p* < 0.01 vs. DB (granulocytes); ns = not significant.

The live-cell assay revealed rapid and significantly increased internalization of NAXI, but not DB, especially in larger PBMCs (12–15 µm in diameter) corresponding to monocytes (Figure 7A). Flow cytometric analyses of whole blood showed that monocytes and granulocytes were GD2^−^ and in part CD64^+^. Lymphocytes were double negative (Figure 7C,D). Accordingly, lymphocytes did neither internalize DB nor NAXI (Figure 7E, left). In contrast, a significantly higher number of monocytes and granulocytes internalized NAXI compared to DB (*p* = 0.0166, NAXI 18 ± 3% and DB 6 ± 1% for monocytes and *p* = 0.0083, NAXI 9 ± 3% and DB 1 ± 2% for granulocytes, Figure 7E, center and right, respectively). These results show a stronger internalization of NAXI compared to DB mainly by CD64^+^ monocytes.

### 3.8. Impact of Soluble GD2 on the NAXI- and DB-Mediated ADCC

**Figure 8:**
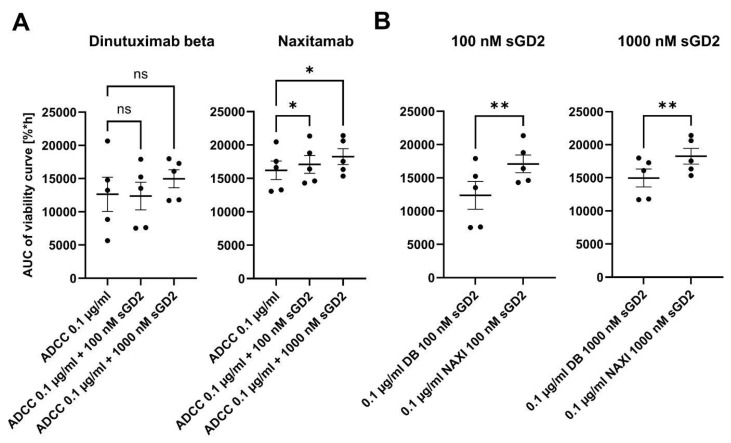
Impact of soluble GD2 (sGD2) on anti-GD2 antibody-mediated cellular cytotoxicity. Spheroids were transduced to yield a stable near-infrared fluorescence for viability tracking. Tumor spheroids were treated with 75,000 PBMCs, 0.1 µg/mL dinutuximab beta (DB), and naxitamab (NAXI) (ADCC) with and without 100 and 1000 nM sGD2 for 168 h. The area under the viability curve (AUC) was calculated for each curve, and the loss of viability results in a decrease of viability values. (**A**) Graph shows the AUC (viability values) of spheroids treated with NAXI, DB, and PBMCs (ADCC) with and without 100 or 1000 nM sGD2. (**B**) Comparison of AUC (viability values) of DB- and NAXI-mediated ADCC in the presence of 100 nM and 1000 nM sGD2. Data are shown as means from at least five independent experiments (done in four replicates) ± SEM. (**A**,**B**) ANOVA with appropriate post hoc test: (**A**) * *p* < 0.05 vs. NAXI ADCC; ns = not significant. (**B**) ** *p* < 0.01.

We investigated the functional implications of the presence of sGD2 for NAXI and DB-mediated ADCC. For this purpose, the CHLA-20 neuroblastoma spheroid viability assay (168 h) was used in the presence and absence of 100 and 1000 nM sGD2, and the area under the viability curve (%*h) was calculated and plotted (Figure 8). The addition of 100 and 1000 nM sGD2 significantly reduced the NAXI-mediated ADCC potency as indicated by higher area under the viability curve values compared to the control. In contrast, sGD2 had no significant effects on DB-mediated ADCC (Figure 8A). Upon direct comparison, DB-mediated ADCC remained significantly higher in the presence of 100 and 1000 nM sGD2 compared to NAXI-mediated ADCC (Figure 8B).

Our results show the direct connection between TMDD, ADCC, and target affinity of the investigated GD2 antibodies.

## 4. Discussion

We evaluated the functional potency of two anti-GD2 antibodies approved for the treatment of high-risk neuroblastoma, NAXI and DB. Although the constant regions of both antibodies were engineered to be of human IgG1 isotype, there are substantial structural differences between both molecules. NAXI is a humanized variant of the murine anti-GD2 antibody 3F8 created by CDR grafting, whereas DB is a human–mouse chimeric variant of the murine anti-GD2 antibody 14.G2A. Although both antibodies are produced in Chinese Hamster Ovary (CHO) cells, suggesting similar post-translational glycosylation patterns, the protein sequences of both molecules are completely different, reflected by a ten-fold higher binding affinity of NAXI to GD2 compared to DB, which influences their function [2].

Our study demonstrates a higher ADCC potency of DB compared to NAXI (Figure 1) attributed to a stronger TMDD effect for NAXI compared to DB (Figure 2, Figure 3, Figure 4, Figure 5, Figure 6, Figure 7 and Figure 8), a phenomenon that has been described for high- versus intermediate-affinity antibodies. The enhancement of the binding affinity of a mAb can lead to trade-offs with other interdependent properties [4], including an increased susceptibility for the antigen sink effect, also known as TMDD [11]. This phenomenon occurs when antibodies bind strongly to abundant target antigens, leading to rapid internalization and degradation of the antibody–antigen complex within cells [11,12,18]. TMDD is also driven by additional mechanisms linked to elevated affinity, such as the binding of soluble target antigens or an increased tendency for the antibody to remain bound, thereby reducing its potential for re-engagement with target cells. As a result, high-affinity antibodies can exhibit nonlinear pharmacokinetics characterized by rapid clearance from the systemic circulation, especially at lower doses [19]. The TMDD effect is more pronounced in conditions with high antigen expression, such as neuroblastoma tumors overexpressing GD2, and shedding of the tumor antigen increases the TMDD effect of a high-affinity antibody [19]. A high sGD2 serum concentration of up to 1000 nM was reported for neuroblastoma patients [17]. In general, intermediate-affinity antibodies are less affected by internalization and degradation, maintain a more stable concentration, and are therefore available to engage immune effector cells more efficiently compared to high-affinity antibodies [11,13,20]. Furthermore, intermediate-affinity antibodies exhibit faster off-rates and may predominantly bind antigens monovalently, thus increasing Fc density on target cells and enhancing immune engagement [9,10,13,21]. In contrast, high-affinity antibodies, which typically bind bivalently (i.e., using both Fab arms), can reduce Fc accessibility and consequently attenuate ADCC responses when compared to intermediate-affinity counterparts [9,10,13].

We could demonstrate a clear role for TMDD likely attributed to the higher affinity of NAXI compared to DB to off-viable tumor GD2 (Figure 4 and Figure 5) as well as to a more rapid internalization of NAXI compared to DB (Figure 6 and Figure 7). This leads to a lower amount of antibody available for GD2 binding to on-viable tumor after exposure to dead GD2-expressing tumor cells (Figure 4), soluble GD2 (Figure 5), or following internalization into tumor- (Figure 6) or immune cells (Figure 7). The extent of this effect was dependent on the tumor spheroid model and most pronounced at lower antibody concentrations (Figure 1 and Figure 5). The internalization may also contribute to the rapid clearance of the antibody from the tumor microenvironment, further exacerbating the TMDD effect and diminishing its ability to mediate ADCC in vivo. Interestingly, even leukocytes, particularly monocytes, showed significantly greater internalization of NAXI than of DB mediated by CD64^+^ cells (Figure 7). This might be due to the Fc engineering of NAXI to improve Fc-FcR affinity, resulting in higher internalization rates [2]. However, the binding signal of NAXI to neuroblastoma cells showed similar or higher gMFI values compared to DB across all concentrations, indicating similar or higher tumor cell surface coverage by NAXI (Figure 3).

We could not show any CDC anti-tumor activity for both antibodies in the 3D spheroid models, which more closely mimic the 3D structure of tumors in vivo in contrast to 2D models (Figure 2). This indicates that ADCC is the most effective mechanism of action, which has also been concluded from clinical trials with DB, where neuroblastoma patients with a high-affinity FcγR polymorphism showed a better outcome [22]. Further evidence for a subordinate role of CDC compared to ADCC for antibody-mediated anti-neuroblastoma immune responses can be concluded from clinical trial data with hu14.18K322A, which is a humanized anti-GD2 antibody generated by CDR grafting using the CDR regions of the anti-GD2 antibody 14G2a. The constant human IgG1 regions were engineered introducing the K322A mutation to reduce complement activation. Despite decreased CDC activity, hu14.18K322A maintained its anti-tumor efficacy while significantly reducing neuropathic pain, a major on-target off-tumor side effect of anti-GD2 antibodies, attributed at least in part to CDC [23]. This supports the notion that ADCC, rather than CDC, is the primary driver of anti-tumor activity of anti-GD2 antibodies. In this context, a potential methodological consideration is the use of mixed PBMC populations instead of isolated NK cells for the ADCC assays. Although NK cells are the principal effectors of anti-GD2 antibody-mediated ADCC via FcγRIIIa (CD16), we opted to use unseparated peripheral blood mononuclear cells (PBMCs) rather than isolated NK cells or neutrophils in our cytotoxicity assays, in line with the established practice for ADCC evaluation [24,25,26]. This decision was based on the intent to preserve a physiologically relevant immune cell context, which better reflects the in vivo situation. PBMCs include not only NK cells, but also monocytes and subsets of T cells that may contribute to or modulate antibody-dependent responses [27]. While isolation of specific effector populations may provide more mechanistic insight, it may also lead to artificial activation or loss of functionality due to processing steps [28,29]. To minimize variability and control for non-specific effects, we included appropriate antibody-free controls in all experiments. Additionally, PBMCs from multiple donors were used in independent replicates, and consistent trends were observed across all datasets. We therefore consider the use of PBMCs to be a valid and informative approach for the assessment of ADCC in this study.

DB is approved for patients with high-risk neuroblastoma in the maintenance phase of newly diagnosed patients as well as for patients with relapsed and refractory disease, and it is given as a continuous infusion over 24 h on 10 consecutive days per cycle for 5 cycles (100 mg/m^2^/cycle; 10 mg/m^2^/day). The long-term infusion was developed because side effects are less intensive and less frequent [30]. This is in contrast to NAXI, which is approved for patients with relapsed and refractory neuroblastoma with osteomedullary disease. It is given as short infusions of 30 min duration on days 1, 3, and 5 of each cycle (9 mg/kg/cycle = 270 mg/m^2^/cycle; 3 mg/kg/day = 90 mg/m^2^/day). The treatment intervals between DB and NAXI cycles are 4–5 weeks for NAXI and 5 weeks for DB. For DB, pharmacokinetic data are available showing trough levels ranging from 0.1 to 1 µg/mL preceding the next cycle, which results in an immunologically active antibody concentration throughout the entire treatment period of approximately six months, as shown here for 3D targets (Figure 1) but also in 2D models [30,31]. For NAXI, pharmacokinetic data are not reported; therefore, it is not clear if the 9-fold higher daily dosing or the 3-fold higher dose per cycle of NAXI compared to DB outweighs the TMDD disadvantage of NAXI compared to DB (Figure 2, Figure 3, Figure 4, Figure 5, Figure 6, Figure 7 and Figure 8). Furthermore, the current practice is to combine NAXI with GM-CSF, whereas DB is given without additional cytokine co-medication [14]. The treatment of neuroblastoma patients with cytokines (IL-2 and GM-CSF) is associated with a strong increase of activated CD64^+^ granulocytes and monocytes [32]. This co-medication may lead to a further increase of the internalization rate and magnitude in particular of mAbs engineered to have a high affinity to the FcR, which may further aggravate the TMDD effect for NAXI.

While our data consistently demonstrate TMDD-driven effects across multiple models, the interpretation of these results should consider potential limitations. This proof-of-concept study relied on human in vitro systems and did not include in vivo mouse experiments. Thus, organism-level pharmacokinetics/biodistribution, continuous-infusion dosing and stromal components (e.g., cancer-associated fibroblasts) were not considered in the models used. Murine xenograft studies using 3F8 have shown potent PBMC/PMN-mediated ADCC and superior tumor control after humanization [2]. Affinity maturation of hu3F8 (NAXI) improved binding (7-13-fold), ADCC (up to 12-fold), and tumor ablation in vivo, but also revealed an “affinity ceiling” beyond which additional gains were minimal [33]. Our data demonstrate TMDD-driven functional loss at equimolar concentrations for clinical-grade NAXI compared to DB. However, the optimal affinity range for GD2 and clinical relevance require confirmation in further model systems and clinical trials. 

## 5. Conclusions

In conclusion, we compared two approved anti-GD2 antibodies (DB and NAXI) for the treatment of high-risk neuroblastoma patients in preclinical models and reported a higher ADCC potency of DB (intermediate GD2 affinity) compared to NAXI (higher GD2 affinity) attributed to TMDD. Further studies are needed to translate these findings into adaptations of current clinical practice. Validation in clinical datasets will be essential to confirm the relevance of these effects.

## Data Availability

The data that support the findings of this study are available from the corresponding author upon reasonable request.

## Supplementary Materials

The following supporting information can be downloaded at: https://www.mdpi.com/article/10.3390/cancers17152510/s1, Figure S1: (A) GD2 expression profile (left) and gMFI of GD2 signals (right) of neuroblastoma cells used for the generation of 3D spheroid models. (B) Examples of spheroid viability over time curves using dinutuximab beta at indicated concentrations in the presence of PBMC (75.000/well) (left); representative fluorescence microscopy images of spheroids, 4× magnification (right). (C) Example and visual representation for the calculation of the area under the viability over time curve; Figure S2: Antibody-dependent cellular cytotoxicity (ADCC) mediated by DB and NAXI against GD2 negative COG-N-519- or SK-N-SH- (A) or GD2 low SHEP-2 spheroids (B).

## References

[1] Ladenstein R., Pötschger U., Valteau-Couanet D., Luksch R., Castel V., Ash S., Laureys G., Brock P., Michon J.M., Owens C. (2020). Investigation of the Role of Dinutuximab Beta-Based Immunotherapy in the SIOPEN High-Risk Neuroblastoma 1 Trial (HR-NBL1). Cancers.

[2] Cheung N.-K.V., Guo H., Hu J., Tassev D.V., Cheung I.Y. (2012). Humanizing Murine IgG3 Anti-GD2 Antibody m3F8 Substantially Improves Antibody-Dependent Cell-Mediated Cytotoxicity While Retaining Targeting In Vivo. Oncoimmunology.

[3] Natsume A., Niwa R., Satoh M. (2009). Improving Effector Functions of Antibodies for Cancer Treatment: Enhancing ADCC and CDC. Drug Des. Devel. Ther..

[4] Rabia L.A., Desai A.A., Jhajj H.S., Tessier P.M. (2018). Understanding and Overcoming Trade-Offs between Antibody Affinity, Specificity, Stability and Solubility. Biochem. Eng. J..

[5] Zahavi D., Weiner L. (2020). Monoclonal Antibodies in Cancer Therapy. Antibodies.

[6] https://ir.ymabs.com/static-files/52dfb61a-bb9a-472e-a6ff-473284a00cf5.

[7] Tang Y., Lou J., Alpaugh R.K., Robinson M.K., Marks J.D., Weiner L.M. (2007). Regulation of Antibody-Dependent Cellular Cytotoxicity by IgG Intrinsic and Apparent Affinity for Target Antigen. J. Immunol..

[8] Opaliński Ł., Szymczyk J., Szczepara M., Kucińska M., Krowarsch D., Zakrzewska M., Otlewski J. (2018). High Affinity Promotes Internalization of Engineered Antibodies Targeting FGFR1. Int. J. Mol. Sci..

[9] Mazor Y., Yang C., Jack Borrok M., Ayriss J., Aherne K., Wu H., Dall’Acqua W.F. (2016). Enhancement of Immune Effector Functions by Modulating IgG’s Intrinsic Affinity for Target Antigen. PLoS ONE.

[10] Mazor Y., Sachsenmeier K.F., Yang C., Hansen A., Filderman J., Mulgrew K., Wu H., Dall’Acqua W.F. (2017). Enhanced Tumor-Targeting Selectivity by Modulating Bispecific Antibody Binding Affinity and Format Valence. Sci. Rep..

[11] An G. (2020). Concept of Pharmacologic Target-Mediated Drug Disposition in Large-Molecule and Small-Molecule Compounds. J. Clin. Pharmacol..

[12] Wang B., Lau Y.Y., Liang M., Vainshtein I., Zusmanovich M., Lu H., Magrini F., Sleeman M., Roskos L. (2012). Mechanistic Modeling of Antigen Sink Effect for Mavrilimumab Following Intravenous Administration in Patients with Rheumatoid Arthritis. J. Clin. Pharmacol..

[13] Oostindie S.C., Lazar G.A., Schuurman J., Parren P.W.H. (2022). Avidity in Antibody Effector Functions and Biotherapeutic Drug Design. Nat. Rev. Drug Discov..

[14] Mora J., Castañeda A., Gorostegui M., Santa-María V., Garraus M., Muñoz J.P., Varo A., Perez-Jaume S., Mañe S. (2021). Naxitamab Combined with Granulocyte-Macrophage Colony-Stimulating Factor as Consolidation for High-Risk Neuroblastoma Patients in Complete Remission. Pediatr. Blood Cancer.

[15] Siebert N., Seidel D., Eger C., Jüttner M., Lode H.N. (2014). Functional Bioassays for Immune Monitoring of High-Risk Neuroblastoma Patients Treated with ch14.18/CHO Anti-GD2 Antibody. PLoS ONE.

[16] Troschke-Meurer S., Zumpe M., Meißner L., Siebert N., Grabarczyk P., Forkel H., Maletzki C., Bekeschus S., Lode H.N. (2023). Chemotherapeutics Used for High-Risk Neuroblastoma Therapy Improve the Efficacy of Anti-GD2 Antibody Dinutuximab Beta in Preclinical Spheroid Models. Cancers.

[17] Balis F.M., Busch C.M., Desai A.V., Hibbitts E., Naranjo A., Bagatell R., Irwin M., Fox E. (2020). The Ganglioside GD2 as a Circulating Tumor Biomarker for Neuroblastoma. Pediatr. Blood Cancer.

[18] Ng C.M., Stefanich E., Anand B.S., Fielder P.J., Vaickus L. (2006). Pharmacokinetics/pharmacodynamics of Nondepleting Anti-CD4 Monoclonal Antibody (TRX1) in Healthy Human Volunteers. Pharm. Res..

[19] Mager D.E., Jusko W.J. (2001). General Pharmacokinetic Model for Drugs Exhibiting Target-Mediated Drug Disposition. J. Pharmacokinet. Pharmacodyn..

[20] Dunlap T., Cao Y. (2022). Physiological Considerations for Modeling in Vivo Antibody-Target Interactions. Front. Pharmacol..

[21] Wang B., Yang C., Jin X., Du Q., Wu H., Dall’Acqua W., Mazor Y. (2020). Regulation of Antibody-Mediated Complement-Dependent Cytotoxicity by Modulating the Intrinsic Affinity and Binding Valency of IgG for Target Antigen. mAbs.

[22] Siebert N., Jensen C., Troschke-Meurer S., Zumpe M., Jüttner M., Ehlert K., Kietz S., Müller I., Lode H.N. (2016). Neuroblastoma Patients with High-Affinity FCGR2A, -3A and Stimulatory KIR 2DS2 Treated by Long-Term Infusion of Anti-GD Antibody ch14.18/CHO Show Higher ADCC Levels and Improved Event-Free Survival. Oncoimmunology.

[23] Furman W.L. (2021). Monoclonal Antibody Therapies for High Risk Neuroblastoma. Biol. Targets Ther..

[24] Mata M.M., Mahmood F., Sowell R.T., Baum L.L. (2014). Effects of Cryopreservation on Effector Cells for Antibody Dependent Cell-Mediated Cytotoxicity (ADCC) and Natural Killer Cell (NK) Activity in 51Cr-Release and CD107a Assays. J. Immunol. Methods.

[25] Strong D.M., Ortaldo J.R., Pandolfi F., Maluish A., Herberman R.B. (1982). Cryopreservation of Human Mononuclear Cells for Quality Control in Clinical Immunology. I. Correlations in Recovery of K- and NK-Cell Functions, Surface Markers, and Morphology. J. Clin. Immunol..

[26] Boero S., Morabito A., Banelli B., Cardinali B., Dozin B., Lunardi G., Piccioli P., Lastraioli S., Carosio R., Salvi S. (2015). Analysis of in Vitro ADCC and Clinical Response to Trastuzumab: Possible Relevance of FcγRIIIA/FcγRIIA Gene Polymorphisms and HER-2 Expression Levels on Breast Cancer Cell Lines. J. Transl. Med..

[27] Wang Z., Chimenti M.S., Strouse C., Weiner G.J. (2021). T Cells, Particularly Activated CD4^+^ Cells, Maintain Anti-CD20-Mediated NK Cell Viability and Antibody Dependent Cellular Cytotoxicity. Cancer Immunol. Immunother..

[28] Blanter M., Cambier S., De Bondt M., Vanbrabant L., Pörtner N., Abouelasrar Salama S., Metzemaekers M., Marques P.E., Struyf S., Proost P. (2022). Method Matters: Effect of Purification Technology on Neutrophil Phenotype and Function. Front. Immunol..

[29] Peng H., Endo Y., Wu W.J. (2024). Define Critical Parameters of Trastuzumab-Mediated ADCC Assays via Assay Optimization Processes, Focusing on the Impact of Cryopreserved Effector Cells on Assay Performance. Cancers.

[30] Lode H.N., Ehlert K., Huber S., Troschke-Meurer S., Siebert N., Zumpe M., Loibner H., Ladenstein R. (2023). Long-Term, Continuous Infusion of Single-Agent Dinutuximab Beta for Relapsed/refractory Neuroblastoma: An Open-Label, Single-Arm, Phase 2 Study. Br. J. Cancer.

[31] Siebert N., Troschke-Meurer S., Marx M., Zumpe M., Ehlert K., Gray J., Garaventa A., Manzitti C., Ash S., Klingebiel T. (2018). Impact of HACA on Immunomodulation and Treatment Toxicity Following ch14.18/CHO Long-Term Infusion with Interleukin-2: Results from a SIOPEN Phase 2 Trial. Cancers.

[32] Troschke-Meurer S., Siebert N., Marx M., Zumpe M., Ehlert K., Mutschlechner O., Loibner H., Ladenstein R., Lode H.N. (2019). Low CD4^+^/CD25^+^/CD127^−^ Regulatory T Cell- and High INF-γ Levels Are Associated with Improved Survival of Neuroblastoma Patients Treated with Long-Term Infusion of ch14.18/CHO Combined with Interleukin-2. Oncoimmunology.

[33] Zhao Q., Ahmed M., Guo H.-F., Cheung I.Y., Cheung N.-K. (2015). Alteration of Electrostatic Surface Potential Enhances Affinity and Tumor Killing Properties of Anti-ganglioside GD2 Monoclonal Antibody hu3F8. J. Biol. Chem..

