# Supplementary material for: Affinity Affects the Functional Potency of Anti-GD2 Antibodies by Target-Mediated Drug Disposition
Source: Cancers (Basel). 2025 Jul 30;17(15):2510. doi: 10.3390/cancers17152510 (PMC12345752; doi:10.3390/cancers17152510)
Supplement: Supplementary file 1 [file cancers-17-02510-s001.zip › cancers-3711562-supplementary.pdf]

Supplemental Figures

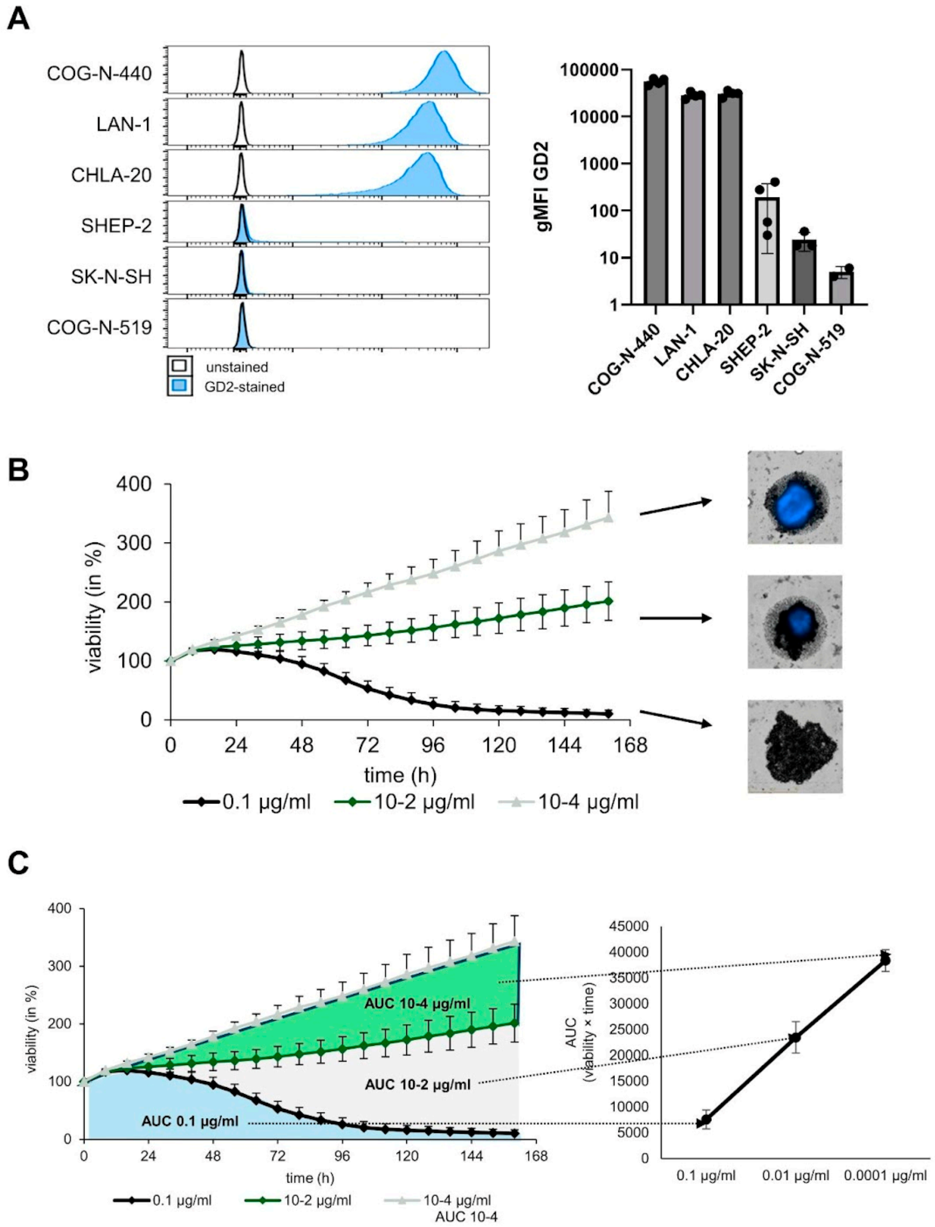

Supplemental Figure S1. (A) GD2 expression profile (left) and gMFI of GD2 signals (right) of neuroblastoma cells used for the generation of 3D spheroid models. (B) Examples of spheroid viability over time curves using dinutuximab beta at indicated concentrations in the presence of PBMC (75.000/well) (left); representative fluorescence microscopy images of spheroids (right). (C) Example and visual representation for the calculation of the area under the viability over time curve.

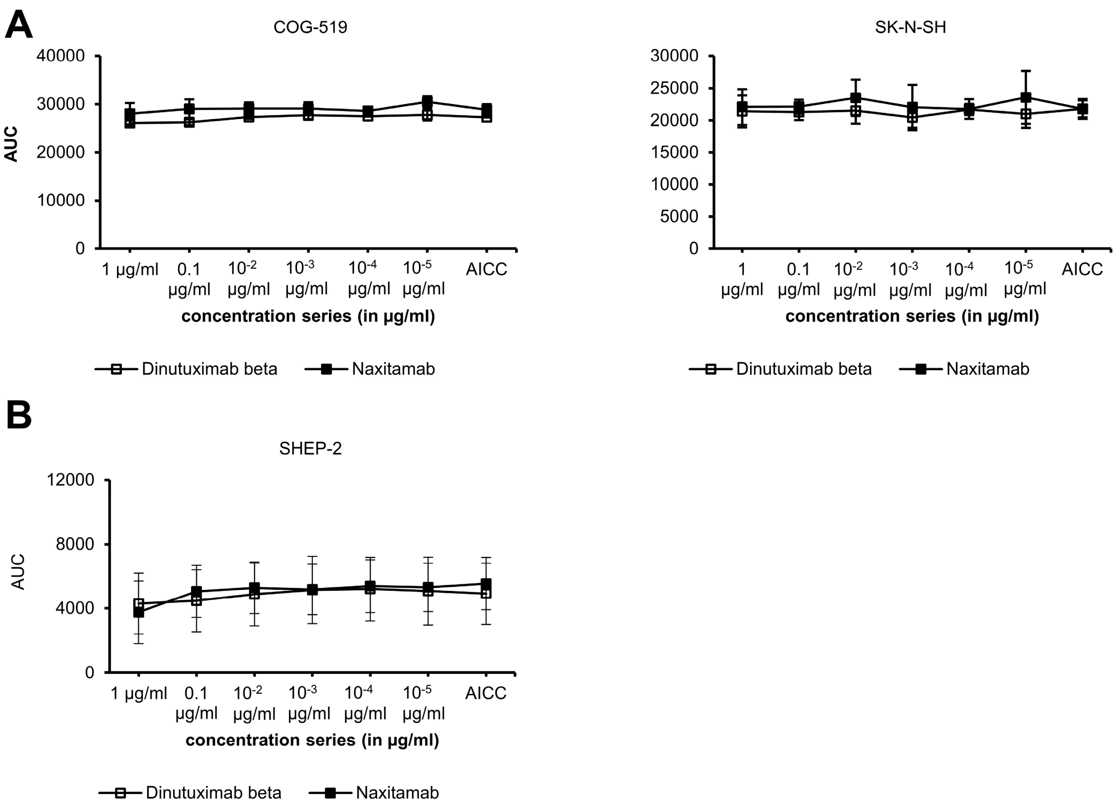

Supplemental Figure S2. Antibody-dependent cellular cytotoxicity (ADCC) mediated by DB and NAXI against GD2 negative COG-N-519- or SK-N-SH- (A) or GD2 low SHEP-2 spheroids (B). The viability of tumor spheroids was monitored using neuroblastoma cells expressing the near-infrared fluorescent protein (iRFP680). Spheroids were treated for 168 h with serial dilutions of the GD2-specific antibodies dinutuximab beta (DB; intermediate affinity) or naxitamab (NAXI; higher affinity), each co-incubated with 75,000 peripheral blood mononuclear cells (PBMCs). As a negative control, spheroids were treated with PBMCs alone (without antibodies) to assess antibody-independent cellular cytotoxicity. Viability at each time point was calculated by dividing the integrated spheroid fluorescence by the fluorescence at baseline (0 h). The graphs depict spheroid area under the curve (AUC) viability at antibody concentrations of 10-5 – 1 µg/ml and the AICC control, as indicated in the legend. Data represent mean AUC values ± SEM from at least five independent experiments, each performed in four replicates. A paired t-test was used to compare DB vs. NAXI AUC values ($p > 0.1$).
